# Developing an Exploratory Framework Linking Australian Aboriginal Peoples’ Connection to Country and Concepts of Wellbeing

**DOI:** 10.3390/ijerph10020678

**Published:** 2013-02-07

**Authors:** Jonathan Kingsley, Mardie Townsend, Claire Henderson-Wilson, Bruce Bolam

**Affiliations:** 1 School of Health and Social Development, Deakin University, Burwood, Victoria 3125 Australia; E-Mails: mardie.townsend@deakin.edu.au (M.T.); claire.henderson-wilson@deakin.edu.au (C.H.-W.); 2 Melbourne School of Population Health, University of Melbourne, Carlton, Victoria 3010 Australia; E-Mail: bbolam@vichealth.vic.gov.au

**Keywords:** wellbeing, Aboriginal, health, Country, nature

## Abstract

Aboriginal people across Australia suffer significant health inequalities compared with the non-Indigenous population. Evidence indicates that inroads can be made to reduce these inequalities by better understanding social and cultural determinants of health, applying holistic notions of health and developing less rigid definitions of wellbeing. The following article draws on qualitative research on Victorian Aboriginal peoples’ relationship to their traditional land (known as Country) and its link to wellbeing, in an attempt to tackle this. Concepts of wellbeing, Country and nature have also been reviewed to gain an understanding of this relationship. An exploratory framework has been developed to understand this phenomenon focusing on positive (e.g., ancestry and partnerships) and negative (e.g., destruction of Country and racism) factors contributing to Aboriginal peoples’ health. The outcome is an explanation of how Country is a fundamental component of Aboriginal Victorian peoples’ wellbeing and the framework articulates the forces that impact positively and negatively on this duality. This review is critical to improving not only Aboriginal peoples’ health but also the capacity of all humanity to deal with environmental issues like disconnection from nature and urbanisation.

## 1. Introduction

In the following paper “Aboriginal” refers to Aboriginal and Torres Strait Islander people as “Aboriginal Victorian” represents Aboriginal people from the state of Victoria. “Indigenous” will refer to Traditional Custodians’ international with “non-Indigenous” describing people who do not identify as the above. The reasons such terminology was chosen include that the National Aboriginal Community Controlled Health Organisation, the peak body for Aboriginal Community Controlled Health Services in Australia recommends this language [1] and in recognition and honor capitals will be applied [2]. Holmes and colleagues [3], who uses Aboriginal in research noted “the terms ‘Aboriginal’ and ‘indigenous’ are problematic because they refer to first nation’s people who have in common the experience of colonisation, but whose experiences are also very different”. 

Aboriginal people of Australia suffer great health inequalities in comparison to their non-Indigenous counterparts [4,5,6,7]. Life expectancy data estimates between a 9.7/11.5 (for females and males respectively) to 17 year gap between Aboriginal and non-Indigenous Australians [5,8,9,10]. It is damning that successive governments have allowed the oldest living culture to suffer such health, social, economic and political inequalities. Aboriginal Victorian people experience the same fate as those in other states, suffering similar health inequalities [11,12]. 

Aboriginal Australians suffer more pervasive social disadvantages and potentially worse health problems than any other Indigenous population in the developed world, with health outcomes comparable to Third World countries [6,7,13]. Evidence indicates that such inequalities can be understood by focusing on factors including colonisation, intergenerational trauma, social and cultural determinants of health and considering holistic notions of wellbeing [4,10,11]. Holistic, throughout this paper, refers to the “interconnection” of determinants impacting physical, social, environmental, emotional, spiritual, psychological and cultural wellbeing of the individual and community [14,15]. Recently, there has been attention placed on social and emotional wellbeing, empowerment and intergenerational trauma measures [5,7,10,16] to capture holistic views of Aboriginal health, but greater understanding and more robust evidence is required. 

Government measures addressing social and emotional wellbeing are still relatively rigid, focusing on extreme cases like psychological distress, happiness, anger, life stresses, social gradient and poverty [6,7,16,17]. The literature suggests that Aboriginal Australians suffer from higher levels of psychological stress, discrimination, distress and trauma compared with other Australians [10,16,17]. Aboriginal health statistics, however, cannot be used effectively without a clearer understanding of these inequalities and what underpins them [7,10]. This paper highlights the need to gather more robust understandings of Aboriginal social determinants of health and wellbeing in order to “Close the Gap” [18,19,20] in health outcomes between Victorian Aboriginal and non-Indigenous people. 

The following article provides a starting point for “Closing the Gap” [21,22] by focusing on Aboriginal definitions of wellbeing. Academics and governments struggle to describe Aboriginal views of wellbeing, reducing it to a matrix of standard socio-economic indicators and bio-medical measures rather than complex Aboriginal concepts which include issues like kinship, connection to Country and the like [5,7,23,24,25,26]. They tend to omit holistic determinants of health, potentially causing suspicion among Aboriginal people of data collection practices and the way health is measured [5,7,24]. 

Taylor [25] and Panelli and Tipa [27] outlined Aboriginal and Indigenous wellbeing as revolving around cultural factors including social relationships, connection to Country, kinship, traditional knowledge, reciprocity, identity, accountability and physical, social, spiritual and emotional wellbeing. It must be recognized that no “one size fits all” definition of wellbeing will work, because each Aboriginal community is unique and such concepts are not static or frozen but are influenced by a combination of past and present factors [5,10,28]. Taylor [25] highlighted that it is critical not to simplify and obscure Indigenous worldviews in order to reduce the complexity of the Aboriginal concept of wellbeing into measurable indicators. Through the development of a framework, this article aims to explicate this holistic concept of wellbeing. The framework draws on literature exploring Aboriginal peoples’ connection to Country and its link to wellbeing, as well as primary qualitative research focused on Aboriginal Victorian peoples’ understanding of this topic by the lead author, under the supervision of the co-authors.

Such a framework accords with the emerging field of ecohealth which acknowledges that “contemporary ecological, health and social sciences have much to learn from the holistic philosophy of Indigenous peoples and their traditional expertise derived from centuries of refining knowledge about the links between ecosystems and health” [29]. Ecohealth is transdisciplinary, linking human health and wellbeing to ecology and ecosystem approaches, identifying the interconnectedness of environmental, socio-cultural and economic factors by applying ecology and public health approaches [30,31,32]. Parkes [33] acknowledged that “ecohealth approaches that address health more holistically, and encourage integration and exchange among multiple forms of knowledge, suggest a new terrain of research and practice that can greatly benefit from, and potentially be highly complementary to, holistic approaches to Aboriginal... health”. 

The framework presented in this paper brings a number of different disciplines together to illuminate an understanding of the significance of contact with the natural world, not only for Aboriginal Victorian communities but more widely, by using a population group still connected with the natural environment. The framework is not about measurement; rather it attempts to move beyond Western models to provide a holistic understanding of Aboriginal Victorian views of connection to Country and to consider the implications of this for wellbeing. 

### 1.1. Understanding Human Wellbeing

Wellbeing is a complex and hard to measure concept [15,34,35]. Wellbeing is often used in place of terms like health, quality of life [34,36] and spiritual and emotional sentiment [37,38]. To understand the underpinnings of wellbeing, one needs to consider each lived experience across the lifespan and the potential for it to affect individuals and communities [38,39,40]. Stewart [15] notes that wellbeing encompasses different “dimensions, including physical, psychological, social, mental and spiritual health”. 

Social and emotional wellbeing, trauma and unhealed loss indicators highlight terrible trends occurring in Aboriginal communities [10]. Preliminary indicators assume that postnatal depression is more frequent for Aboriginal compared with non-Aboriginal women due to this trauma, colonsiation and socio-economic inequalities [26]. Aboriginal people reported that 77% of close friends/family had experienced at least one “life stressor” in the last twelve months [17]. Further, 27% of Aboriginal Australians reported high to very high levels of psychological stress [16]. Aboriginal Victorian peoples’ had a one in four chance of high to very high psychological distress levels, almost twice that of non-Indigenous Victorians [41]. This data indicates that Aboriginal Victorian peoples’ are at high risk of suffering from anxiety and depression, with 35% percent diagnosed with these disorders compared to 20% of non-Indigenous Victorians [41].

By better understanding the underpinnings of wellbeing, we can aim to reduce these rates of ill-health in Aboriginal populations. Furnass [39] and Trewin [40] perceived wellbeing as a critical measure of improve health and noted that contributing factors include the interaction of satisfactory human relationships, meaningful occupation, income, contact with nature, creative expression and social arrangements. Research indicates that the effects of income and socio-economic status on individuals’ wellbeing is substantial [42], while Diener and Seligman [43] and Shields and Wheatley Price [44], noted that although economics is central other areas like social environment, positive and negative emotions, and “social contingent aspects of psychological wellbeing are statistically important”. Research highlights that social status and environmental conditions also play a key role in human wellbeing [45,46,47,48,49,50,51]. 

A positive development in this field relates to subjective well-being (SWB) because it assesses multiple measures [52,53,54,55,56]. SWB is a way of measuring wellbeing by assessing individuals’ life experiences, considering both affect and cognition, sense of happiness and evaluation of life based on feelings, moods and emotions [43,47,55,57,58]. Diener and Chan [56] highlighted high levels of SWB (associated with variables like life satisfaction, happiness and optimism) may improve health outcomes. Literature clearly identifies “that family, friendship, trust… security, meaningful work, and a sense of purpose are all important correlations of subjective wellbeing” [59]. Diener *et al.* [58] mentioned SWB research is required in the exploration of cross-cultural approaches and environmental factors. Initial research has found that there are differences in how cultures perceive SWB variables but there is “some universal causes of well-being and ill-being” [60]. 

Indigenous concepts of wellbeing are viewed as “more holistic” than most Western constructs, adopting a whole-of-life view that “align[s] spiritual, social, and cultural elements in intimate connection with biophysical bases” [28]. Dockery [23] and Prout [24] identified elements of wellbeing included cultural health like kinship, social networks, and reciprocal relationships with Country. Destructions and disconnection from Country cause major distress for Aboriginal people [61]. The gap in understanding of the Indigenous view of wellbeing is demonstrated in most definitions, especially by governments who compartmentalize elements into separate areas of measurement [25]. Taylor [25] noted “Indigenous peoples’ perceptions and understanding of well-being extend beyond, and sometimes conflict with, many of the indicators currently adopted by global reporting frameworks”. The understanding of wellbeing in this paper draws on a number of determinants that impact on the individual throughout the whole-of-life using Country as a central theme.

### 1.2. Understanding Country (and Western Notions of Nature)

“In Western society, we rest comfortably on our inherent truths about the nature of nature. Our burgeoning environmental literature... contains a nearly endless variety of statements about the absence of mind of nature. The environment is numb to a human presence...

We may be elevated by the beauty of nature, cling to it, crave to protect it; but we cleave to the coldness of stone, the storm that carries us away without knowing, the waters that kill without reason. We live alone in an uncaring world of our creation” [62].

It seems fitting that Aboriginal communities refer to their traditional lands and territories through more enmeshed language like “Country” or “homelands”. Country refers to everything including the land, air, water and stories of “Dreaming”, being dynamic and multilayered, forming the rules, norms and beliefs of existence between species and humans through connecting Aboriginal peoples’ back to ancestral beings from the time of creation [63,64]. This is evident in birthing practices linking Aboriginal women to their kinship and Country [14,26]. Weir [65] highlighted that “in country humans and nature, and nature and culture, are not regarded as separate, but are entangled together in all types of relationships”. Aboriginal people have little room to separate themselves from Country as they are embedded within it. Such a spiritual connection has been built over thousands of generations of responsibilities and knowledge attached to its management [66]. McKnight [67] acknowledges that Country:

“Constitutes identity, and loss of land is tantamount to loss of one’s self… To have one’s own Country is to have a place where one can withdraw in times of trouble and where one can easily find sustenance… it bestows a degree of independence that cannot otherwise be obtained.”

In contrast, Western concepts of nature, like wellbeing, are broad and at times contradictory terms [68,69,70,71]. Soper [69] defined nature as everything that is not human or “artificially worked or produced”, but debated this statement, saying that “humanity is a component of nature” in which the two cannot be separated. Western definitions commonly refer to nature as being marginalized from society and needing to be controlled, often viewed as female in respect of dominant power relations, or as a constructed structure, over time, becoming part of nature [70]. Strang [72] noted that “nature is imagined as “the other”, there is a perceptual separation between social and environmental sustainability… this separation leads to a crucial distancing... of nature and—inevitably—to unsustainable environmental relationships.” Weir [65] reiterated that “the influence of separating humans from nature has also contributed to a rationalist and utilitarian approach to country”.

However, researchers should not over romanticize Aboriginal peoples’ connection to land, which has changed over time, is complex and not rooted in the past [27,72]. It should be acknowledged that “Indigenous and non-Indigenous views about country are not mutually exclusive… However, there is a marked differences in Indigenous peoples’ spiritual connection to place. This connection, created through extremely long relationships… is expressed through belief systems and knowledge of country” [66]. This Aboriginal knowledge has occurred through extensive experience, passed down through storytelling, songs, and mythology explaining ancestral paths [64]. Taking into account these themes, a working definition of Country and nature will be: viewing and/or actively interacting with features of the biophysical environment that provides spiritual, cultural, historical or emotional meaning.

### 1.3. Country and Wellbeing

Indigenous peoples hold a deep connection to their ancestral landscapes being central to their wellness [29,73,74,75,76,77,78]. Connection to Country is central to Aboriginal peoples’ existence [26,79] and reflects the notion of an innate connection to land [62]. Disconnection from this land “compromises cultural connections” and causes extreme distress and powerlessness commonly felt by many Indigenous groups worldwide [61,78,80]. Just as lack of control, stress and social gradient have been shown to effect peoples’ health outcomes across all population groups [81] this disconnect from Country may contribute an extra layer to this distress and inequality. There is extensive evidence acknowledging the health and wellbeing benefits of Aboriginal land management programs which is viewed as a method of addressing health inequalities [75,82]. Kingsley and colleagues [83] have highlighted the links between Victorian Aboriginal peoples’ connection to Country and their existence, articulated by a participant saying:

“you can put your trust in the land because it is your ancestors; you know it’s going to guide you in the right direction and protect you…”

John Clarke [84], a Victorian Aboriginal park ranger, pointed out that no matter if you are Aboriginal or not, all people have a role to play in the management of Country and feel this deep spiritual connection. Environmental narratives exploring these connections play a fundamental role in people’s everyday wellbeing, however, they are still often overlooked in scientific arenas [85,86]. Kingsley and colleagues [83] suggested that local Aboriginal environmental narratives and knowledge should be accorded similar standing to scientific information. 

Indeed, scientific information provides us with a similar picture, highlighting the wellbeing benefits of contact with nature, with disconnection from nature impacting detrimentally on human happiness and ecology [87,88,89,90]. Evidence identifies the psychological benefits of horticulture and gardening, indicating that such activities have a protective role by improving human wellbeing [71,91,92,93]. Purely being in the outdoors can be effective in strengthening wellbeing for vulnerable populations [94,95,96]. Research by Frumkin [97,98] and Guite *et al.* [99] looked at environments which promote people’s health and wellbeing and identified green open spaces playing a key role in this process. 

Wellbeing has been a catch phrase in ecology since the UN Millennium Ecosystem Assessment (2001) identified nature as a critical pathway to enhancing quality of life [100]. Destruction of natural environments has long been considered of detriment to individuals’ wellbeing acknowledged in the Ottawa Charter of Health Promotion (1986), especially for Aboriginal communities [101,102,103,104,105,106] whereas environmental management in local neighborhoods has contributed to increased wellbeing [87,107,108,109]. Studies have made the link between improved subjective wellbeing and contact with nature [89]. Put simply by Mayer and colleagues [87]:

“For 350,000 generations humans have lived close to land as hunter gathers… it would be surprising if the modern life of being divorced from nature did not have some negative consequences associated with it and that being in nature had positive benefits.”

Lockwood’s [110] examination of intrinsic values of the environment identifies that humans’ view nature “above” themselves when they engage in the natural world. People who engage in the natural environment are known to have a greater appreciation for protecting what they perceive as biodiversity and stronger attitudes towards ecological activism [90,111,112,113,114,115]. Due to urbanisation, people’s main form of connecting back to nature is through parks, gardens, pets and public nature reserves. Humans’ inherent connections to nature have been disconnected and dislocated [116]. Therefore, there does seem to be a great deal to learn from the connection Aboriginal people have to Country, if we are to improve wellbeing for all humanity.

## 2. A Review of Models

A number of theories, narratives and models explaining humans’ connection to nature have been produced, with little unity between them. However, researchers are beginning to develop more comprehensive holistic frameworks and explanations of human-nature relationships [117,118,119]. Waltner-Toews [118] highlighted the fact that “theoretical development provides the basis for the generalization of sustainable action... the specifics of what forms that may take, especially for health practitioners, have yet to be brought together in a coherent way”. He explains that the best way to collect “good scholarly inquiry” on ecohealth theories is to gather qualitative information and stories to develop comprehensive frameworks and tools [118]. This approach was applied in this paper by reviewing qualitative data linking Aboriginal Victorian peoples’ wellbeing to connection to Country, in order to develop a new framework. 

**Figure 1:**
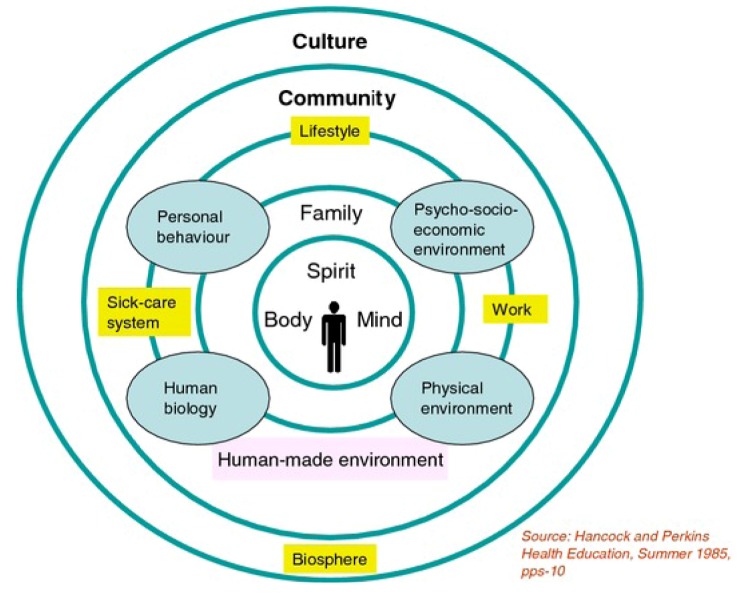
The Mandala of Health [120].

In presenting this framework, it is critical to highlight some models used in the past to explain such links. Health practitioners have started to move away from a purely individual responsibility for health, common in mainstream biomedicine, and are looking toward an ecological model of health that focuses more on the wellbeing experienced associated with where one lives. This view was popularized after the holistic “Mandala of Health” model was developed (Figure 1). 

The four major factors affecting populations in this model are human biology, personal behavior, psychosocial environment and the physical environment, with this model emphasizing the intertwining of natural science, medical science and social science in influencing the health of individuals and communities [120,121,122].

**Figure 2:**
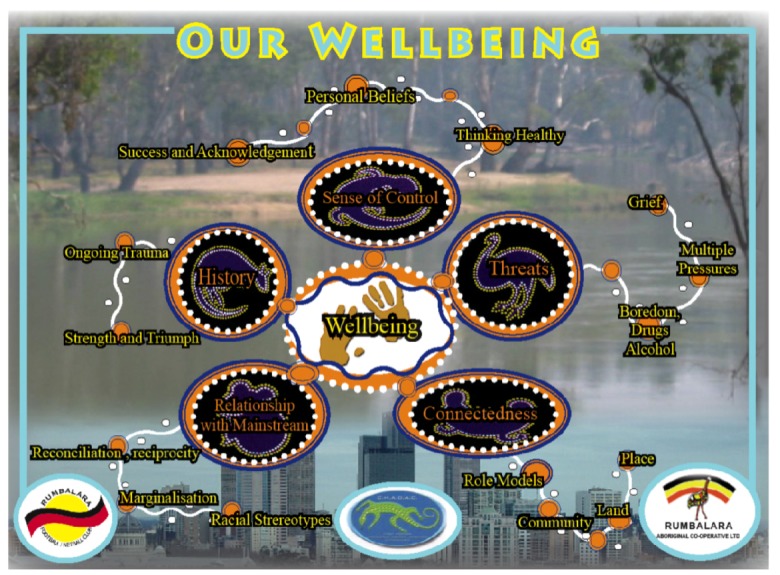
“Our Wellbeing” A holistic model of Indigenous Wellbeing [124].

Aboriginal Community Controlled Health Organizations (ACCHOs) have been at the forefront of incorporating holistic and culturally appropriate models into practice in primary health care settings in Australia [10,123]. The following model was developed by Rumbalara Aboriginal Cooperative [124], an ACCHO in Victoria. This model focuses on the interrelated nature of wellbeing and the environment (Figure 2). It explores broad elements that affect Aboriginal peoples’ wellbeing, such as connectedness, sense of control, and history, and visually shows how these factors interrelate. An important element of this model is the background, with the top showing Barmah Forest, the Country that Rumbalara Aboriginal Cooperative members identify with and, down the bottom, Melbourne city. This symbolizes the conflict Aboriginal Victorian people find themselves in, being urbanized but wanting to still connect to their Country [125]. 

**Figure 3:**
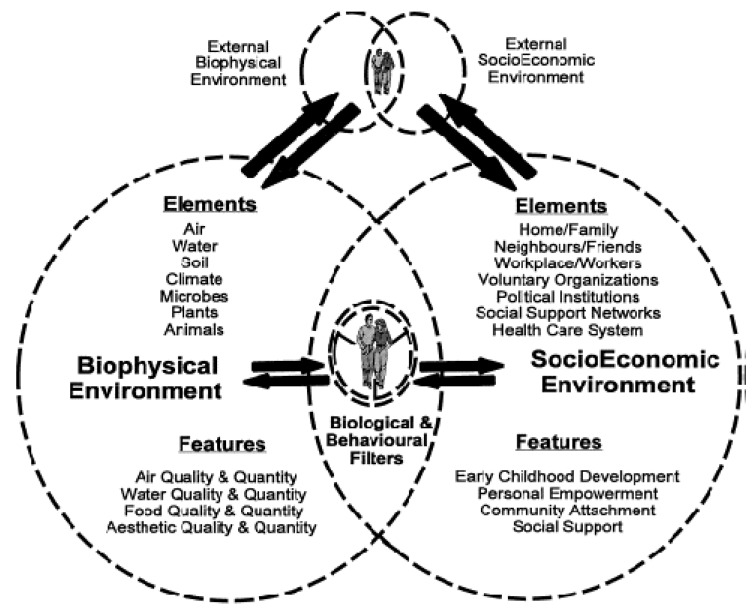
Butterfly Model of Health for an Ecosystem Context [117].

Another holistic model which influenced the lead author’s development of the new framework was VanLeeuwen and colleagues’ [117] “Butterfly Model of Health for an Ecosystem Context” (Figure 3) which focused on internal and external “Socio Economic Environments” and “Biophysical Environments” factors surrounding “Biological and Behavioural Filters”. This model identifies determinants which impact on one’s life. Although this model has clear links to wellbeing, it omits language identifying this term.

## 3. Discussion

**Figure 4:**
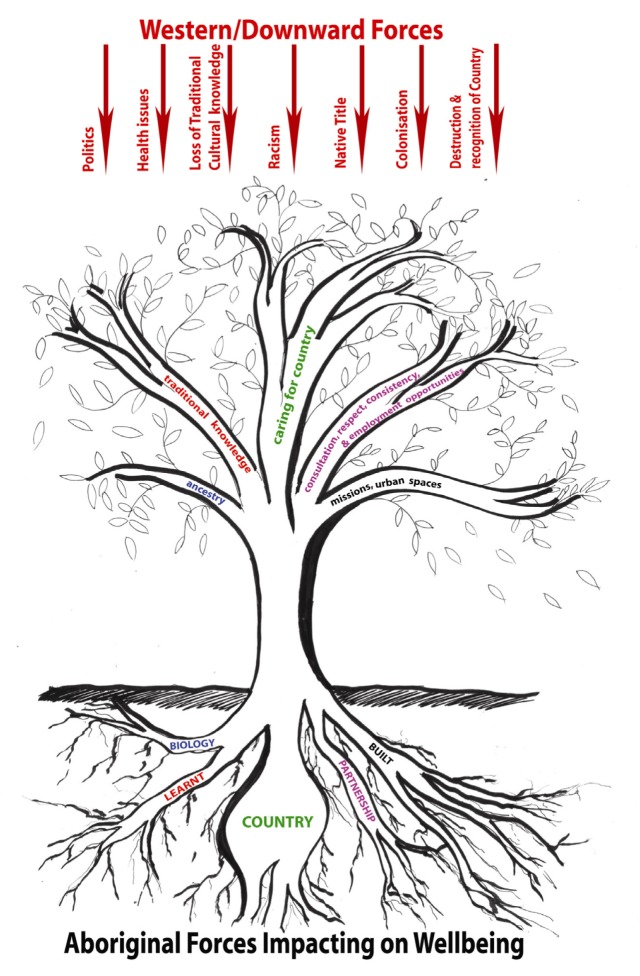
Exploratory Framework for Aboriginal Victorian peoples’ wellbeing.

The following framework (Figure 4) is based on eight years of research conducted by the lead author exploring connections between Aboriginal peoples’ wellbeing and connection to Country and on a review of the literature. The exploratory framework is aimed to provide a better visual understanding of Aboriginal peoples’ connection to/interaction with Country. Such interaction is not in isolation from, but rather involves, a number of interrelated factors, which will be referred to as forces. These forces influence Aboriginal Victorian peoples’ wellbeing positively and negatively. The information from which this framework was derived was from qualitative semi-structured interviews with three Traditional Custodian groups and Aboriginal land managers in Victoria, so provides only a snapshot of the story, even though including an extensive literature review.

This framework depicts multiple forces affecting wellbeing. The framework thus aims to reflect the holistic nature of wellbeing, including physical, social, emotional, political, cultural and environmental influences [126,127,128]. In Figure 4, the tree, referred to as “Aboriginal Forces Impacting on Wellbeing”, reflecting social, cultural, environmental and economic determinants of health, which positively impact on the Aboriginal Victorian population. The roots represent the fundamental components of wellbeing and the branches build on these roots, reflected in the linking colors. “Western/Downward Forces” reflect elements that impede Aboriginal Victorian peoples’ ability to improve their wellbeing, which are pushing down on this symbolic tree. What the framework tries to depict is that these forces are competing and not complementary, causing an imbalance in wellbeing. 

### 3.1. Aboriginal Forces Impacting on Wellbeing

Research [83] identified the role of Country in strengthening Aboriginal Victorian peoples’ self-esteem, self-worth and pride, fostering self-identity and belonging, cultural and spiritual connection, enabling positive states of wellbeing and acting like a sanctuary to escape pressures. As one informant emphasized, caring for Country and knowledge around it ensures wellbeing:

“If the land’s healthy, the animals are healthy, it makes the people healthy … but if the animals don’t have habitat than that means the land and people are sick” [83].

“Learnt experiences” refer to how such knowledge and practices of caring for Country are passed down. This knowledge base has been passed down over thousands of generations to Aboriginal Victorian people and therefore is tested, credible and a reliable source of information on environmental management [129,130]. This learnt knowledge builds on Van Leeuwen and colleagues [117] “Butterfly Model of Health for an Ecosystem Context” “Socio-Economic Environments” section components, such as “Early Childhood Development” and “Personal Empowerment”. However, this knowledge is more complex and spiritual, being passed down through creation stories, narratives, sacred names, ceremonies, art and dance.

“Biology” is also evident in the “Human Biology” component in the Mandala of Health, which refers to one’s genetics [120]. In contrast, biology for Aboriginal Victorians loosely refers to passing down of ancestry through connection to Country. This is the basis of the biophilia hypothesis, which acknowledges humans have been connected to the land for thousands of generations, causing the brain to be hardwired innately to such bonds [89,131,132]. This study [83] reiterated that this ancestral connection back to Country was automatic and allowed Aboriginal Victorians to feel a sense of belonging, obligation and spiritual connectedness to care for their territories. The clearest biophilic connection in the study came from a Traditional Custodian, part of the stolen generation, who commented: 

“Identity from the land remained within me and when I did my art it’s symbolic and [the] Elders have picked up on the old scenes. I think there’s something printed in our DNA which has ancestral memory” [83].

Often scholars will associate the built environment with urban places [116,133] but for this paper, this term has to be put in the Aboriginal Victorian context. Since colonisation, Aboriginal Victorian people were placed in missions with many sacred sites becoming desecrated, urbanised and tamed; but over time these spaces have “evolved into the [positive] identity… of Aboriginal people”, symbolising the resilience and strength of the culture [134]. Missions are considered symbolic of this resilience today; however, historically, missions have had negative connotations associated with discriminatory government policies. This is reflected in the “Western/Downward Forces” section of the framework. 

The overturning of this negative history would not be possible without “Partnerships”, collaboration and trust built over time between the Aboriginal and non-Indigenous population. Building strong relationships, consultation processes, education and training pathways, reciprocity and respect, and employment opportunities were identified as key to these collaborative partnerships working. It was noted that if they (the relationships and opportunities available to Aboriginal Victorians) are not involved in caring for Country programs, they are destined to fail [125,136,137]. This is supported in the literature, which identifies collaboration, funding, education, cultural exchange, capacity building, trust and respect are fundamental in fostering Aboriginal land management projects [138,139,140].

### 3.2. Western/Downward Forces

Aboriginal Victorian people live in “two worlds”—one recognizing their rich culture, and the other (defined in the proposed framework as “Western downward forces”) denying it [11,125]. Kingsley and colleague [125] and Barra and colleagues [141] noted that government employees were viewed as having different values and a lack of understanding of Aboriginal community members. Aboriginal government employees in Victoria identified that bureaucracy was “flawed, and not in touch with Aboriginal communities” [136] as they excluded communities, were too focused on reporting and poorly coordinated between government departments. This meant Aboriginal Victorian communities would speak to numerous government departments about similar issues. The lack of understanding of Aboriginal culture has blurred power structures, causing communities and families, at times, to be pitted against each other [125,136]. This is referred to as “Politics” in the figure. Such “Politics” is evident with a Traditional Custodian acknowledging:

“The saddest thing is we are fighting over boundaries when we are never going to get Native Title… before we were united just fighting for recognition” [125].

“Native Title” was seen as divisive in Victoria because it pitted groups against each other and disappointed communities not successful in gaining land. Native Title aims to recognise Aboriginal sovereignty of their continued relationship with Country through Australian Law [125] Although there have been inroads with this policy recently (for example the Native Title successes in 2007 of the Gunditjmara people and in 2010 of the Gunai/Kurnai people) there is a long process of healing in Victoria that will need to happen for Aboriginal communities to be recognized as Traditional Custodians. Lack of recognition and access to Country is considered an injustice for Aboriginal people and has been found to cause health issues [125,137]. The poor health outcomes (referred to as “Health Issues”) evident in Aboriginal populations was identified by some researchers [125,136] as causing cultural knowledge of Country to be lost because information was not being passed on to community members through observation, training and practice, as it once was, thus causing fragmentation. This has led to “Loss of Traditional Cultural Knowledge”.

“Racism” has also caused much distress in Aboriginal Victorian communities, leading to negative stereotypes. Aboriginal Victorian people often mentioned feeling excluded and not valued, with racism “ingrained into society” [125,136,137]. Aboriginal Victorian people interviewed wanted recognition of their Traditional Custodian status and knowledge, rather than having non-Indigenous people believing they knew everything about Aboriginal culture and, as a consequence, making them feel marginalised. In part this is due to the cycle that has been occurring since “Colonisation”, which had a devastating impact on the Aboriginal culture in Victoria due to politicians and bureaucrats making decisions for the community [125]. All these factors have affected Aboriginal peoples’ ability to be effectively involved in the workforce, land management and education system. Coupled with the destruction of the natural environment over the last 200 years, this means Aboriginal Victorian peoples today need to work harder to reconnect to Country with all these forces at play. This is symbolized in the figure as “Destruction & Recognition of Country”.

## 4. Conclusions

This article aimed to provide insight of how Aboriginal Victorian peoples connect to Country, providing a framework for exploring this connection. This framework aimed to move beyond conventional wellbeing models which are often rigid. This is of importance in the field of ecohealth as, by having an understanding of this holistic approach to contact with Country, we can apply it with other populations to improve collaborative understandings of forces which impact on wellbeing.

Kingsley and colleagues [83] noted that Aboriginal peoples may have a more realistic view to explain their connection to their natural world than Western concepts. Aboriginal Victorian people identified that this connection goes beyond words and is steeped in spiritual orientation to that locality. Therefore, the rationale for designing a framework was to give greater recognition to this connection so that it can cut across different research fields more effectively. The process of reviewing wellbeing literature and its link to the human-nature relationship was fundamental in gaining an understanding of this meaning. 

The framework, its surrounding evidence base and applied model, could be a starting point for future application and debate. At a time where humanity is facing fundamental issues such as dangerous climate change, increased urbanisation and disconnection from the natural environment, the development of such a framework is timely. To effectively tackle such issues, academics, policy makers and communities as a whole must unite around common understandings of these concepts and ideas. 

What this process aimed to achieve is to bring together years of research and apply it to develop a framework in order to tackle future environmental and health issues. This allows the moving away from often complex theoretical constructs or static guidelines into a space where this concept can be understood by a wider audience. Specialists working in the public health or environmental fields could apply this framework to advocate why being connected to the local natural environment is critical to people’s wellbeing. It is the authors’ opinion that by greater interaction in the natural environment, there will be greater momentum to protect such areas and therefore improve population health and ecology. 
